# Median Pancreatectomy for Benign and Low-Grade Malignant Pancreatic Lesions: An 11-Year Single-Center Experience With Perioperative and Functional Outcomes

**DOI:** 10.7759/cureus.115326

**Published:** 2026-08-27

**Authors:** Hariharan Ramesh, Ajith Vettuparambil, Sajith K Mohan, Mahesh S, Lekha Vijayalekshmi, John Mathew Manipadam, Amal Goutham, Yelisetti Vamsee Priya, Jazeel S A, Govind Purushothaman

**Affiliations:** 1 Gastrosurgery, Lakeshore Hospital and Research Centre, Cochin, IND

**Keywords:** central pancreatectomy, median pancreatectomy, pancreatic fistula, pancreatic function preservation, pancreatic neoplasms, parenchyma preserving pancreatic surgery

## Abstract

Background

Median pancreatectomy (MP) is a parenchyma-sparing procedure for benign and low-grade malignant lesions of the pancreatic neck and body, with the potential to preserve endocrine and exocrine function. However, concerns regarding postoperative pancreatic fistula (POPF) have limited its widespread adoption.

Methods

This retrospective cohort study included 23 consecutive patients who underwent MP at a tertiary referral centre between January 2015 and December 2025. POPF was classified using the 2016 International Study Group of Pancreatic Surgery criteria, and long-term endocrine and exocrine function was assessed during follow-up.

Results

The mean age was 43.57 ± 15.05 years, and 91.3% of the patients were female. Common indications included cystic neoplasms (60.9%), neuroendocrine tumours (21.7%), and solid pseudopapillary neoplasms. Biochemical leak occurred in 60.9% of patients, and Grade B POPF occurred in 30.4%. Patients with Grade B POPF had a significantly longer hospital stay than those with biochemical leak (25.00 ± 15.41 vs. 14.29 ± 7.53 days, p = 0.039). No mortality was observed. At a median follow-up of 98 months (range, 4-130 months), only one patient (4.3%) developed new-onset diabetes, and none developed exocrine insufficiency.

Conclusion

MP is a safe and effective parenchyma-sparing procedure with excellent long-term preservation of pancreatic function, despite the high incidence of biochemical leak and Grade B POPF, which can generally be managed conservatively with appropriate clinical care.

## Introduction

Median pancreatectomy (MP), also known as central or middle pancreatectomy, is a parenchyma-sparing surgical approach for benign, borderline, and low-grade malignant lesions located in the neck and proximal body of the pancreas. The reconstructive concept was first described by Guillemin P and Bessot M in 1957 in a patient with chronic calcifying pancreatitis [1, 2], and the complete procedure was later performed by Dagradi A and Serio G for an insulinoma of the pancreatic neck [3]. Unlike conventional resections such as pancreaticoduodenectomy or distal pancreatectomy, MP preserves both the pancreatic head and tail, theoretically maintaining maximal endocrine and exocrine function [4].

The rationale for MP is compelling. Although pancreaticoduodenectomy is oncologically sound for malignant lesions, it carries significant morbidity and the potential for long-term metabolic complications [1]. Distal pancreatectomy, although technically simpler, sacrifices substantial pancreatic parenchyma and frequently necessitates splenectomy, with reported rates of new-onset diabetes ranging from 7% to 40%, depending on the extent of resection and underlying pancreatic pathology [5]. In contrast, MP aims to resect only the diseased segment while preserving healthy pancreatic tissue on both sides of the lesion.

Despite these theoretical advantages, MP has not gained widespread acceptance in pancreatic surgery. The primary concern relates to the two pancreatic transection surfaces, which create two potential sites for leakage and may increase the risk of postoperative pancreatic fistula (POPF) [6,7]. Early series reported POPF rates ranging from 25% to 59%, significantly higher than those associated with conventional pancreatectomy [8-10]. This elevated fistula rate, combined with the technical complexity of the procedure, has limited MP to highly selected cases in specialized centres.

However, recent advances in perioperative management, the standardization of POPF classification through the International Study Group on Pancreatic Surgery (ISGPS) criteria [6], and enhanced surgical techniques have renewed interest in parenchyma-sparing procedures. Several contemporary series have demonstrated that, although POPF rates remain elevated, most fistulas can be managed conservatively [11-13]. Furthermore, the long-term functional benefits of preserving pancreatic parenchyma may outweigh the short-term morbidity associated with POPF.

The increasing detection of benign and borderline pancreatic lesions through the widespread use of cross-sectional imaging has created a growing population of patients who may benefit from function-preserving surgery [14,15]. These patients, who are often young and otherwise healthy, face decades of potential metabolic consequences following extensive pancreatic resection. For such individuals, MP offers an attractive alternative that balances oncological adequacy with quality-of-life considerations.

India has witnessed the gradual adoption of MP, with several centres reporting their experiences [16-18]. In parallel, larger international series have consistently demonstrated the feasibility of MP and highlighted its long-term functional advantages, providing the foundation for its increasing acceptance in carefully selected patients [19,20]. The published literature from the Indian subcontinent remains limited, with most series comprising fewer than 20 patients and having relatively short follow-up periods [21-24]. Long-term data on the preservation of endocrine and exocrine function, which represents the primary justification for this technically demanding procedure, are particularly scarce.

This study presents an 11-year single-centre experience with MP, focusing on perioperative outcomes, POPF incidence and severity, and the long-term preservation of pancreatic function.

## Materials and methods

This retrospective cohort study was conducted at Lakeshore Hospital and Research Centre, Kochi, Kerala, India, a high-volume tertiary referral centre with a dedicated multidisciplinary pancreatic surgery unit. The study included all 23 consecutive adult patients who underwent median (central) pancreatectomy between January 2015 and December 2025. The primary objective was to evaluate perioperative outcomes, postoperative complications, and the long-term preservation of endocrine and exocrine pancreatic function following MP. Institutional ethics approval was obtained before the commencement of the study, and patient confidentiality was maintained in accordance with institutional policies and the Declaration of Helsinki.

Patients who underwent MP for benign, borderline, or low-grade malignant pancreatic lesions were included in the study. Electronic medical records were systematically reviewed using a standardised data collection proforma. Demographic variables included age, sex, comorbidities, and pre-existing diabetes mellitus. Clinical variables included presenting symptoms, duration of symptoms, incidental versus symptomatic presentation, lesion size, anatomical location within the pancreas (neck, proximal body, or body), pancreatic duct diameter, preoperative imaging findings, utilisation of endoscopic ultrasound (EUS), and preoperative biochemical investigations. Operative variables included the surgical approach (open or laparoscopic-assisted MP) and parenchymal resection techniques. Histopathological findings, including the final diagnosis and tumour characteristics, were recorded for all patients.

The decision to perform MP was made intraoperatively after complete exploration and assessment of the pancreatic lesion. MP was selected for lesions confined to the pancreatic neck or proximal body when adequate proximal and distal pancreatic parenchyma could be preserved and an oncologically appropriate resection was considered feasible. Patients with extensive vascular involvement, multifocal disease, or lesions requiring formal pancreaticoduodenectomy or distal pancreatectomy were excluded from MP. Intraoperative frozen-section examination of the pancreatic transection margin was performed whenever indicated to confirm margin negativity, and additional resection was undertaken, if required, to achieve an R0 resection.

Following complete mobilisation of the pancreatic neck and body, the proximal pancreatic stump was divided using a stapler fitted with a black cartridge and applied using a graded approximation technique [ECHELON FLEX™ (Ethicon, Inc., Cincinnati, OH, USA) or Signia™ system (Medtronic, Minneapolis, MN, USA)]. In selected cases in which stapler application was not feasible, the proximal pancreas was transected sharply and closed with interrupted non-absorbable sutures after secure ligation of the main pancreatic duct. The distal pancreatic remnant was transected sharply, the main pancreatic duct was identified and cannulated when feasible, and reconstruction was performed using duct-to-mucosa pancreaticojejunostomy or invagination pancreaticojejunostomy, depending on the duct size and gland texture. The pancreaticojejunostomy was performed using fine, absorbable monofilament sutures, with meticulous approximation of the pancreatic capsule to the jejunal seromuscular layer.

A 24-Fr Foley catheter was routinely placed adjacent to the pancreatic transection site as an intra-abdominal drain following MP. Drain output was recorded daily, and drain fluid amylase (DFA) levels were assessed according to the institutional postoperative protocol. Drain removal was considered when the 24-hour drain output was less than 30 mL and the drain fluid had a low amylase concentration, consistent with the absence of a clinically significant POPF. In patients with persistently high drain output or elevated DFA levels suggestive of a POPF, the abdominal drain was retained at discharge. These patients were instructed to maintain a daily drain output chart and were followed up regularly in the outpatient clinic. Serial downsizing of the drain was performed using progressively smaller infant feeding tubes as the drain output decreased and DFA levels normalised. Complete drain removal was undertaken only after sustained low drain output and resolution of pancreatic leakage. Additional imaging with contrast-enhanced computed tomography (CECT) or USG was performed selectively in patients with clinical suspicion of an intra-abdominal fluid collection, such as persistent fever, abdominal pain, leukocytosis, or failure of drain output to diminish, to guide further management, including percutaneous drainage, if indicated.

Postoperative outcomes were assessed using standardised international definitions. The primary postoperative endpoint was the occurrence and severity of POPF, classified according to the 2016 ISGPS criteria [6]. DFA values measured on postoperative days 1, 3, and 5 were documented and correlated with the development and severity of POPF. Other postoperative complications, including intra-abdominal collections, post-pancreatectomy haemorrhage (PPH), sepsis, pneumonia, melena, subacute intestinal obstruction, and mortality, were recorded. The length of hospital stay was calculated from the date of admission to the date of discharge. Long-term follow-up data were obtained from outpatient clinic visits, inpatient records, laboratory investigations, and telephone follow-up when required. Long-term endocrine function was assessed by documenting the development of new-onset diabetes mellitus or worsening glycaemic control following surgery, whereas exocrine function was evaluated based on the requirement for pancreatic enzyme replacement therapy and clinical evidence of pancreatic exocrine insufficiency. The overall duration of follow-up was calculated from the date of surgery until December 31, 2025.

Statistical analysis was performed using IBM SPSS Statistics, version 20. Continuous variables were assessed for normality using the Shapiro-Wilk test. Normally distributed continuous variables were expressed as the mean ± SD, whereas non-normally distributed variables were reported as the median with IQR. Categorical variables were summarised as frequencies and percentages. Comparisons were made between patients with a biochemical leak and those with grade B POPF. Associations between clinicopathological variables and the occurrence or severity of POPF were evaluated using appropriate univariate analyses. All statistical tests were two-tailed, and a p-value <0.05 was considered statistically significant.

## Results

Patient characteristics

A total of 23 patients underwent MP during the study period. The mean age was 43.6 ± 15.1 years, and the cohort was predominantly female (91.3%). Cystic neoplasm (60.9%) was the most common preoperative diagnosis, followed by neuroendocrine tumour (21.7%) and solid pseudopapillary epithelial neoplasm (SPEN) (17.4%). Nearly half of the patients (47.8%) had no documented comorbidities, whereas diabetes mellitus (34.8%) and hypertension (30.4%) were the most frequently reported coexisting conditions. Baseline demographic and clinical characteristics are summarised in Table 1.

**Table 1:** Baseline demographic and clinical characteristics of the study population (n = 23).

Baseline demographic and clinical characteristics	Mean ± SD or n (%)
Age, years	43.57 ± 15.05
BMI, kg/m²	23.09 ± 3.09
Sex	
Female	21 (91.3)
Male	2 (8.7)
Preoperative diagnosis	
Cystic neoplasm	14 (60.9)
Neuroendocrine tumour (NET)	5 (21.7)
Solid pseudopapillary epithelial neoplasm (SPEN)	4 (17.4)
Comorbidities	
Diabetes mellitus	8 (34.8)
Hypertension	7 (30.4)
Bronchial asthma	1 (4.3)
Coronary artery disease	1 (4.3)
Cerebrovascular accident	1 (4.3)
Dyslipidaemia	1 (4.3)
Non-Hodgkin lymphoma	1 (4.3)
Transient ischaemic attack	1 (4.3)
No documented comorbidity	11 (47.8)

Clinical presentation, operative findings, and postoperative outcomes

Fourteen patients (60.9%) presented with symptoms, whereas nine (39.1%) were diagnosed incidentally. Abdominal pain was the predominant presenting symptom (60.9%), followed by vomiting (30.4%). The mean duration of symptoms was 2.3 ± 3.0 months, and the mean tumour size was 3.4 ± 1.8 cm. Most lesions were located in the pancreatic neck or proximal body (69.6%), and preoperative EUS was performed in 78.3% of patients. Open MP was performed in 17 patients (73.9%), whereas six (26.1%) underwent a laparoscopic-assisted procedure. Reconstruction of the distal pancreatic remnant was performed using duct-to-mucosa pancreaticojejunostomy in 21 patients (91.3%) and invagination pancreaticojejunostomy in two patients (8.7%).

According to the 2016 ISGPS classification, biochemical leak occurred in 14 patients (60.9%), Grade B POPF occurred in seven (30.4%), and no patient developed Grade C POPF. POPF alone constituted the most common postoperative complication (56.5%), while associated complications, including intra-abdominal collection (17.4%), sepsis (4.3%), melena (4.3%), PPH (4.3%), pneumonia (4.3%), supraventricular tachycardia (4.3%), and subacute intestinal obstruction (4.3%), occurred infrequently. There was no postoperative mortality. Clinical presentation, operative details, and postoperative outcomes are shown in Table 2.

**Table 2:** Clinical presentation, tumour characteristics, and perioperative variables of the study population (n = 23). BL: Biochemical leak; DFA: Drain fluid amylase; EUS: Endoscopic ultrasound; ISGPS: International Study Group of Pancreatic Surgery; POPF: Postoperative pancreatic fistula; PPH: Post-pancreatectomy haemorrhage; SAIO: Subacute intestinal obstruction; SVT: Supraventricular tachycardia.

Clinical presentation, tumour characteristics, and perioperative variables	Value
Symptoms	
Incidental	9 (39.1)
Symptomatic	14 (60.9)
Presenting complaint	
Abdominal pain	14 (60.9)
Nausea	1 (4.3)
Vomiting	7 (30.4)
Weight loss	1 (4.3)
Incidental detection	9 (39.1)
Duration of symptoms (months), mean ± SD	2.30 ± 2.95
Tumour size (cm), mean ± SD	3.37 ± 1.81
Length of hospital stay (days), mean ± SD	17.2 ± 11.3
Follow-up duration (months), median ± IQR	98 months (IQR: 4-130)
Location	
Neck and proximal body	16 (69.6)
Body	7 (30.4)
EUS performed	
No	5 (21.7)
Yes	18 (78.3)
Main pancreatic duct	
Non-dilated	19 (82.6)
Dilated	4 (17.4)
Type of surgery	
Open median pancreatectomy	17 (73.9)
Laparoscopic-assisted median pancreatectomy	6 (26.1)
Proximal pancreatic transection	
Sutured	4 (17.4)
Stapled	17 (73.9)
Suture closure with omentoplasty	2 (8.7)
Reinforcement of the proximal stump with Prolene suture	
No	11 (47.8)
Yes	10 (43.5)
POPF	
BL	14 (60.9)
Grade B	7 (30.4)
No POPF	2 (8.7)
Postoperative complications	
None	2 (8.7)
POPF	13 (56.5)
POPF + intra-abdominal collection	4 (17.4)
POPF + sepsis	1 (4.3)
POPF + melena	1 (4.3)
POPF + PPH + SVT + pneumonia	1 (4.3)
POPF + SAIO	1 (4.3)

Histopathological findings and pancreatic endocrine function

On final histopathological examination, macrocystic serous cystadenoma was the most common specific histological diagnosis, accounting for 48.0% of patients and falling within the broader preoperative category of cystic neoplasms, which comprised 60.9% of the cohort. Other lesions included neuroendocrine tumours (21.6%), SPEN (17.5%), an epithelial cyst of the pancreas, a mucinous cystadenoma, and a serous oligocystic adenoma. Histopathological findings are summarised in Table 3.

**Table 3:** Histopathological characteristics of resected pancreatic lesions and comparison of endocrine status before and after median pancreatectomy. SPEN: Solid pseudopapillary epithelial neoplasm.

Histopathological findings	Frequency, n (%)
Macrocystic serous cystadenoma	11 (48.0)
Solid pseudopapillary epithelial neoplasm (SPEN)	4 (17.5)
Neuroendocrine tumour	5 (21.6)
Epithelial cyst of the pancreas	1 (4.3)
Mucinous cystadenoma	1 (4.3)
Serous oligocystic adenoma	1 (4.3)
Endocrine status before and after surgery	
Preoperative endocrine status	
Non-diabetic	16 (69.6)
Diabetic	7 (30.4)
Postoperative endocrine status	
Non-diabetic	15 (65.3)
Patients requiring intensification of therapy	2 (8.6)
Patients who remained diabetic with stable glycaemic control	5 (21.7)
Patient who developed new-onset diabetes	1 (4.3)

Before surgery, 16 patients (69.6%) were non-diabetic, and seven (30.4%) had pre-existing diabetes mellitus. During follow-up, only one patient (4.3%) developed new-onset diabetes mellitus 62 months after MP. Among patients with pre-existing diabetes, glycaemic control remained stable in five patients who continued taking the same oral hypoglycaemic agents (OHAs) after surgery, while two patients required intensification of antidiabetic therapy, either by increasing the dosage of OHAs or by initiating insulin therapy. No patient developed pancreatic exocrine insufficiency requiring enzyme replacement therapy (Table 3).

Factors associated with POPF

Patients who developed Grade B POPF had a significantly longer hospital stay than those with biochemical leak (mean, 25.0 ± 15.4 vs. 14.3 ± 7.5 days; median, 20 (IQR, 14-43) vs. 11 (IQR, 9-18) days; p = 0.039). Age and DFA concentrations on postoperative days 1, 3, and 5 did not differ significantly between the two groups (all p > 0.05) (Table 4).

**Table 4:** Comparison of clinical and biochemical variables according to the severity of postoperative pancreatic fistula (POPF). POPF was classified according to the 2016 International Study Group of Pancreatic Surgery (ISGPS) criteria. BL: Biochemical leak; DFA: Drain fluid amylase; n: Number of patients; POD: Postoperative day; POPF: Postoperative pancreatic fistula.

Variable	POPF	n	Mean	SD	Median (IQR)	p-value
Age	BL	14	41.64	13.45	46 (28.5-52.0)	0.332
	Grade B	7	44.86	20.02	53 (34-60)	
Length of hospital stay	BL	14	14.29	7.53	11 (9-18)	0.039
	Grade B	7	25	15.41	20 (14-43)	
DFA, POD 1	BL	14	8799.79	6941.27	7994.5 (2607.25-14740.25)	0.391
	Grade B	7	55647.71	115237.35	9348 (3186-36528)	
DFA, POD 3	BL	14	5166.86	5438.18	3072.5 (1403.75-7530.50)	0.233
	Grade B	7	36899.29	81742.95	5627 (2862-14736)	
DFA, POD 5	BL	14	7645.14	14984.66	2214 (418.25-5457.25)	0.332
	Grade B	7	23487.71	53187.45	3689 (959-6800)	

None of the evaluated clinicopathological variables, including sex, comorbidities, tumour location, presenting symptoms, operative approach, proximal pancreatic stump management, or stump reinforcement, were significantly associated with the severity of POPF (all p > 0.05). Similarly, neither incidental versus symptomatic presentation nor the method of proximal pancreatic transection influenced the occurrence of Grade B POPF. Details of the univariate analysis are presented in Table 5.

**Table 5:** Univariable analysis of factors associated with the severity of postoperative pancreatic fistula. BL: Biochemical leak; EUS: Endoscopic ultrasound; MP: Median pancreatectomy; POPF: Postoperative pancreatic fistula. Only patients with a biochemical leak or Grade B POPF were included in this analysis; the two patients without POPF were excluded.

Variable	BL, n	BL, %	Grade B, n	Grade B, %	p-value
Sex					
Female (n = 19)	13	68.4	6	31.6	1
Male (n = 2)	1	50	1	50	
Comorbidities					
Yes (n = 11)	7	63.6	4	36.4	1
No (n = 10)	7	70	3	30	
Symptoms					
Incidental (n = 7)	5	71.4	2	28.6	1
Symptomatic (n = 14)	9	64.3	5	35.7	
Location					
Neck and proximal body					0.638
Yes (n = 14)	10	71.4	4	28.6	
No (n = 7)	6	54.5	5	45.5	
Body					
Yes (n = 7)	4	57.1	3	42.9	
No (n = 14)	10	71.4	4	28.6	
EUS performed					
Yes (n = 16)	11	68.8	5	31.3	1
No (n = 5)	3	60	2	40	
Type of surgery					
Laparoscopic-assisted MP (n = 6)	4	66.7	2	33.3	0.845
Open MP (n = 15)	10	66.6	5	33.4	
Proximal pancreatic transection					
Sutured (n = 3)	3	100	0	0	0.318
Stapled (n = 16)	9	56.3	7	43.8	
Suture closure with omentoplasty (n = 2)	2	100	0	0	
Reinforcement of the proximal stump					
No (n = 11)	6	54.5	5	45.5	0.361
Yes (n = 10)	8	80	2	20	

## Discussion

This study represents one of the longest follow-up series of MP reported from India, with a median follow-up of 98 months (range, 4-130 months). Our findings demonstrate that MP is a safe and effective parenchyma-sparing procedure that achieves excellent long-term preservation of endocrine and exocrine function despite a high incidence of POPF.

The demographic profile of our cohort, with 91.3% of patients being female and a mean age of 43.57 years, is consistent with the known epidemiology of benign and borderline pancreatic lesions. Cystic neoplasms and neuroendocrine tumours constituted the majority of indications, reflecting appropriate patient selection for this parenchyma-sparing approach. This distribution aligns with international series in which similar pathologies account for the majority of MP cases [7,9,13].

The most significant finding of our study is the remarkably low rate of new-onset diabetes following MP. Only one patient (4.3%) developed diabetes during the follow-up period, and no patient developed clinically significant exocrine insufficiency. This finding strongly supports the fundamental rationale for MP: preservation of pancreatic parenchyma translates into preserved metabolic function. Our results compare favourably with the 0-7.7% new-onset diabetes rates reported in other large MP series [11-13,19] and contrast sharply with the 25-40% diabetes rates reported after distal pancreatectomy [5,20]. Kang CM et al. demonstrated a statistically significant difference in new-onset diabetes between central pancreatectomy (CP) and extended distal pancreatectomy (0% vs. 31.8%, p < 0.05) [20], corroborating our findings. This functional preservation is particularly relevant for young patients with benign lesions who face decades of potential metabolic complications following extensive pancreatic resection.

The incidence of POPF in our series was 91.3%, comprising 60.9% biochemical leaks and 30.4% Grade B fistulas. Although this rate appears high, it should be interpreted in the context of contemporary literature, which reports POPF rates of 25-59% following MP [7-13,25]. Adham M et al. reported an 8% pancreatic anastomotic leak rate in their series of 50 patients; however, their definition predated the standardised ISGPS criteria [26]. In our study, routine assessment of DFA on postoperative days 1, 3, and 5, together with the application of the 2016 ISGPS classification, may have facilitated the identification of clinically silent biochemical leaks that could have been under-recognised in earlier series using non-standardised definitions. Importantly, most detected leaks were biochemical and did not result in clinically significant morbidity. Although Grade B fistulas required active management, all were successfully managed conservatively with prolonged drainage, antibiotics, and nutritional support. Thus, despite the high overall incidence of POPF, the clinical impact was limited, with most cases representing biochemical leaks without significant morbidity.

The clinical significance of POPF severity is underscored by our finding that Grade B POPF was associated with a significantly longer hospital stay than biochemical leak (25.00 ± 15.41 vs. 14.29 ± 7.53 days, p = 0.039). This represents a definite impact on healthcare resource utilisation and patient recovery. However, the absence of Grade C POPF and zero mortality in our series demonstrate that even clinically relevant fistulas can be managed effectively without catastrophic outcomes. This contrasts with the 1.9-2.5% mortality rates reported in some large MP series [12,13], although most contemporary series report zero or near-zero mortality [11,19,26].

DFA levels on postoperative days 1, 3, and 5 showed a trend towards higher values in patients with Grade B POPF, although these differences did not reach statistical significance. This finding suggests that, although DFA monitoring is useful for early fistula detection, absolute values may not reliably predict fistula severity. Our analysis of risk factors for POPF severity revealed no significant associations with patient demographics, comorbidities, tumour location, preoperative evaluation methods, or surgical approaches. This finding aligns with literature suggesting that POPF after MP is primarily related to the technical aspects of managing two pancreatic transection surfaces in soft, normal pancreatic parenchyma with non-dilated ducts [9,10].

Our study has several limitations. The retrospective design and relatively small sample size limit the statistical power to detect associations between potential risk factors and outcomes. The 11-year study period spans an era of evolving surgical techniques and perioperative management protocols, introducing potential temporal bias. As a single-centre experience from a tertiary referral hospital, our results may not be generalisable to all practice settings. Despite these limitations, our study provides valuable long-term data supporting the role of MP in selected patients with benign and borderline lesions of the pancreatic neck and body. The excellent preservation of endocrine and exocrine function over a median follow-up of 98 months suggests that the short-term morbidity associated with POPF may be offset by durable long-term functional benefits. Future directions should include prospective, multicentre studies comparing MP with alternative resection strategies and incorporating quality-of-life assessments and detailed metabolic evaluations.

## Conclusions

MP is a safe and effective parenchyma-sparing procedure for benign, borderline, and low-grade malignant lesions of the pancreatic neck and body. POPF remains common, but most cases represent biochemical leaks and are clinically inconsequential, while Grade B fistulas can be managed conservatively. The absence of mortality and the low rate of new-onset diabetes (4.3%) over a median follow-up of 98 months support the rationale for this procedure. For appropriately selected patients, particularly young individuals with benign pathology, MP offers a balance between oncological adequacy and quality of life, supporting its continued use in specialised centres with expertise in pancreatic surgery.
